# Evaluation of clinical and laboratory markers of cardiometabolic risk in overweight and obese children and adolescents

**DOI:** 10.6061/clinics/2017(01)07

**Published:** 2017-01

**Authors:** Heloísa Marcelina da Cunha Palhares, Adriana Paula da Silva, Daniela Cristina Silva Resende, Gilberto de Araújo Pereira, Virmondes Rodrigues-Júnior, Maria de Fátima Borges

**Affiliations:** I Divisão de Endocrinologia, Faculdade de Medicina, Universidade Federal do Triângulo Mineiro, Uberaba/MG, Brazil; II Divisão de Enfermagem e Educação Comunitária, Faculdade de Medicina, Universidade Federal do Triângulo Mineiro, Uberaba/MG, Brazil; III Divisão de Imunologia, Faculdade de Medicina, Universidade Federal do Triângulo Mineiro, Uberaba/MG, Brazil

**Keywords:** Cardiometabolic Risk, Children, Adolescents, Overweight, Obesity

## Abstract

**OBJECTIVE:**

This study analyzed the frequency of cardiometabolic risk markers and metabolic syndrome occurrence in overweight and obese children and adolescents.

**METHODS:**

The participants included 161 overweight (n=65) and obese (n=96) individuals aged between 5 and 19 years. Clinical markers were assessed (body mass index, body fat percentage, waist circumference, acanthosis, systolic and diastolic blood pressures, laboratory parameters [glucose, insulin, cholesterol (total and fractions) and triglyceride levels and homeostasis model assessment of insulin resistance (HOMA-IR) index] and leptin and adiponectin levels). The frequency of changes, odds ratios and correlations among markers were determined. Metabolic syndrome was assessed according to International Diabetes Federation criteria.

**RESULTS:**

A high frequency of acanthosis (51.6%); increased waist circumference (45.4%), systolic blood pressure / diastolic blood pressure (8.1% / 9.3%), glucose (10%), insulin (36.9%) and HOMA-IR (44.3%) values; and reduced high-density lipoprotein levels (47.2%) were observed. Leptin levels were increased in 95% of obese and in 66% of overweight subjects. Adiponectin was decreased in 29.5% of obese and in 34% of overweight subjects. An odd ratio analysis revealed a greater probability of increased waist circumference (9.0), systolic blood pressure (4.1), triglyceride (2.3) and insulin (2.9) levels and HOMA-IR (3.0) in the obese group than in the overweight group. The clinical and laboratory parameters and leptin levels exhibited significant correlations, whereas adiponectin was negatively correlated with systolic blood pressure. The occurrence rate of metabolic syndrome was 13.6%.

**CONCLUSIONS:**

The high frequency of changes in clinical, laboratory and adipokine markers indicates the need for early interventions aimed at preventing cardiometabolic complications in adulthood.

## INTRODUCTION

Childhood obesity is widely distributed in developed and developing countries, and the prevalence of excess body weight has increased progressively in the past 30 years, thus becoming an important public health problem worldwide 1. According to data from the World Obesity Federation, 29.5% of all female and 29.7% of all male children and adolescents exhibited excess body weight, including obesity, in the United States between 2013 and 2014 2. In Brazil, data from the Family Budget Surveys (Pesquisa de Orçamentos Familiares – POF) from 2008 to 2009 show that 33.5% of children between 5 and 9 years of age and 20.5% of adolescents aged 10 to 19 years exhibited excess body weight, among whom 14.3% and 4.9% were classified as obese, respectively 3. The Southeast region of Brazil exhibited the highest assessments of excess body weight, namely, 39% in children and 22.6% in adolescents 3.

Obesity in childhood and adolescence is associated with a series of comorbidities, including dyslipidemia, systemic hypertension and changes in glucose metabolism, which in concert with abdominal obesity, define metabolic syndrome. The persistence of obesity is an important risk factor for the development of type 2 diabetes mellitus (DM) and cardiovascular disease (CVD) in adults 1,4,5.

Insulin resistance is an important condition that is associated with overweight and obesity, and studies have indicated that affected individuals exhibit a higher predisposition for the development of metabolic disorders in adulthood 1,6. An excess of body fat not only results in metabolic changes in adipose tissue and the induction of insulin resistance but also leads to endothelial dysfunction via pro-inflammatory and pro-thrombotic effects due to the action of pro-inflammatory cytokines and adipokines 7. Leptin and adiponectin, which are the primary adipokines produced by adipose tissue, play important roles in the cardiometabolic outcomes associated with excess body weight 7,8.

In Brazil, studies assessing cardiometabolic risk markers in overweight children and adolescents show high frequencies of changes in waist circumference (WC), blood pressure, dyslipidemia, insulin resistance and adipokine levels 6,9,10,11.

The search for effective measures to identify obese pediatric patients at a higher risk of developing metabolic and cardiovascular complications constitutes a promising strategy in the prevention of the comorbidities of early obesity 6. Therefore, the objective of the present study was to analyze the family history of excess body weight as well as the clinical, laboratory and adipokine markers of cardiometabolic risk and the occurrence of metabolic syndrome in overweight and obese children and adolescents recruited from public schools in a population study in the city of Uberaba, Minas Gerais, Southeast Brazil.

## MATERIALS AND METHODS

The present work is a descriptive and analytical prospective cross-sectional study that considered a population of 364 overweight and obese children and adolescents (5 to 19 years of age) from public schools of the Municipality of Uberaba, Minas Gerais, Brazil, who had participated in a previous study on the prevalence of overweight status and obesity 12. A total of 161 children and adolescents agreed to participate in the study.

To achieve the proposed objectives, the clinical history of the respective parents, such as the presence of excess body weight, DM, hypertension and dyslipidemia, was obtained by means of an inquiry.

### Clinical Markers

Clinical markers were determined by assessing the nutritional status of the subjects, which included weight, height, skinfolds and WC, and by calculating the body mass index (BMI) using standardized procedures 13,14. According to the criteria proposed by the World Health Organization (WHO, 2006) 13, the nutritional state of all the participants was classified based on the BMI z-score (z-BMI), which was calculated with WHO-Anthro Plus 2007 software, Geneva, Switzerland. Individuals were categorized as overweight (+1 ≤ z-BMI < +2) or obese (z-BMI ≥ +2). The body fat percentage (BFP) was calculated using the sum of four skinfolds as the criterion as in the equations of Slaughter et al. 14, who measured triceps and subscapular skinfold thicknesses. WC was measured while the individual was in a standing position, midway between the lowest rib and the top of the iliac crest, at the end of a normal expiration, with a non-extensible tape measure with millimeter graduations. WC was considered high when the value was within or above the 90^th^ percentile (P90) according to sex and age 15. Physical examinations also included a detailed evaluation of the skin with respect to clinical signs of insulin resistance, manifested by the presence of acanthosis nigricans, which was assessed visually in the neck, armpits and groin.

Systolic and diastolic blood pressures (SBP and DBP, respectively) were measured in the right arm, and the obtained values were classified according to sex, age and height following the criteria of the I Guideline for Preventing Atherosclerosis in Childhood and Adolescence (I Diretriz de Prevenção da Aterosclerose na Infância e Adolescência) of the Brazilian Society of Cardiology 16.

The pubertal stage was evaluated by a single researcher and classified into pre-pubertal and pubertal, according to the criteria of Marshall and Tanner 17,18.

### Laboratory and Adipokine markers

Blood samples were collected after 10 h to 12 h of fasting via peripheral venipuncture to assess the levels of fasting glucose, insulin, total cholesterol, high- and low-density lipoprotein cholesterol (HDL-C and LDL-C, respectively), triglycerides (TGs), leptin and adiponectin. Specifically, serum levels of total cholesterol, HDL-C and TG were assessed by an enzymatic colorimetric assay, and fasting glucose levels were determined by a hexokinase enzymatic assay. All samples were processed in a COBAS 6000-analyzer module C501 (Roche-Hitachi). Serum LDL-C levels were calculated with the equation of Friedewald et al. 19. Insulin levels were measured by electrochemiluminescence (ECL) (COBAS 6000 analyzer, module C601, Roche-Hitachi). Finally, leptin levels were measured in duplicate by an enzyme-linked immunosorbent assay (ELISA) using a commercial kit from EMD Millipore, Millipore Corporation (Billerica, MA, USA), with reference values considered as a BMI of 18 to 25 kg/m**^2^**, i.e., 3.70 to 11.1 ng/mL for women and 2.0 to 5.6 ng/mL for men. Adiponectin levels were measured by an ELISA, employing a commercial kit provided by Abcam Inc. (Cambridge, USA) and expressed as ng/mL.

Metabolic changes were considered according to the I Guideline for Preventing Atherosclerosis in Childhood and Adolescence 16.

The homeostasis model assessment of insulin resistance (HOMA-IR) index was calculated by multiplying fasting plasma insulin (µU/mL) by fasting glucose (mmol/L), divided by 22.5. The cutoff point was ≥2.5 for pre-pubertal 20 and ≥3.43 for pubertal individuals of both sexes 21. The TG/HDL-C ratio (TG/HDL-C) was calculated by dividing the serum TG level by the serum HDL-C level.

### Characterization of metabolic syndrome

Metabolic syndrome was characterized according to the criteria proposed by the International Diabetes Federation (IDF) 22. Regarding the age range <10 years, the criteria of the IDF were adapted, i.e., metabolic syndrome was considered as a WC≥P90 associated with two of the following criteria: fasting glucose ≥100 mg/dL, TG≥130 mg/dL, HDL-C<45 mg/dL and SBP or DBP≥P95, according to sex, age and height.

### Statiscal Analysis

The data were subjected to a descriptive analysis based on absolute and percent frequencies and measures of centrality and dispersion.

The Kolmogorov-Smirnov test was used to analyze the normal distribution of numeric variables, and the homogeneity of variances between the groups was assessed with Levene’s test.

In comparisons between two independent groups, Student’s *t*-test was used for data with a normal distribution and homogeneity of variances, and the Mann-Whitney *U* test was used for data that did not meet the aforementioned criteria.

Categorical variables were compared using the Chi-squared (χ^2^) test, and the odds ratio (OR) was calculated to evaluate the probability of a specific event occurring over another.

Correlations were analyzed with Pearson’s or Spearman’s tests, and multiple linear regression was used for the dependent variables leptin and adiponectin with clinical and laboratory parameters as the predictor variables.

The level of significance was set at 5%. Statistical analyses were performed with the software STATISTICA, Statsoft version 10 and R.

### Ethical Aspects

The present study was approved by the Human Research Ethics Committee of the Federal University of Triângulo Mineiro (Universidade Federal do Triângulo Mineiro), under the number 2479, and all parents/legal guardians signed the informed consent form.

## RESULTS

Regarding the nutritional assessment of the studied population, 40.4% (65/161) were overweight, and 59.6% (96/161) were obese children and adolescents. Among the parents, 65.8% had excess body weight, 10.4% had DM, 32.5% had systemic arterial hypertension, and 19.5% had dyslipidemia.

In the overweight group, the mean age was of 12.6±3.4 years; 70.8% (46/65) were female, and 73.8% (48/65) were pubertal. In the obese group, the mean age was of 10.9±2.5 years; 59.4% (57/96) were female, and 51% (49/96) were pubertal (Table 1). The overweight group had a significantly higher mean age (*p*=0.0002) and predominance of pubertal individuals (*p*=0.003) compared to the obese group. Furthermore, the obese group exhibited significantly higher BMI (kg/m^2^), z-BMI, BFP and WC values than the overweight group (*p*<0.0001), while there was no statistically significant difference between the groups in the mean SBP and DBP measurements (Table 1).

The biochemical, hormone and adipokine profiles of the overweight and obese groups are described in Table 1. The obese group exhibited significantly higher serum total cholesterol, LDL-C, TG, TG/HDL-C, insulin and HOMA-IR values. Regarding adipokines, serum leptin levels were significantly higher in the obese group and were above the adopted reference values in 95% of the obese group and 66% of the overweight group. Adiponectin was not detected in the serum of all of the studied individuals, given that concentrations were below the detection limit of the applied method in 34% of the overweight group and 29.5% of the obese group, with no statistically significant difference between the groups (Table 1).

In the total sample (n=161), the WC was increased (≥P90) in 73 (45.4%) individuals, and the SBP and DBP were increased(≥P95)in 13 (8.1%)and 15 (9.3%)individuals, respectively (Table 2).

Acanthosis nigricans was present in 51.6% of the total sample (83/161) and was significantly higher in the obese group (62/96, 64.6%) than in the overweight group (21/65, 32.3%) (*p*<0.0001; Table 2). The obese group also exhibited a significantly higher percentage of cervical (61.5% *versus* 18.5%) and axillary acanthosis (46.9% *versus* 21.5%) than the overweight group (Table 2).

According to the OR analysis, the probability of increased WC was 9.0 times higher (4.1-19.4) in the obese group than in the overweight group, while the probability of increased SBP was 4.1 times higher (0.9-19.0) and that of the presence of acanthosis nigricans was 3.8 (2.0-7.4) times higher in the obese group (Table 2).

Changes in the laboratory parameters with respect to the reference data ranged from 10.0% (regarding glucose) up to 47.2% (regarding HDL-C) of the studied population. There was a significantly higher ratio of obese children and adolescents with changes in TG (29.2%; *p*=0.04), insulin (46.3%; *p*=0.002) and HOMA-IR index (54.7 %; *p*=0.001) (Table 3).

The OR analysis revealed a 2.3 (1.0-5.0) times greater probability of exhibiting increased levels of serum TG, a 2.9 (1.4-5.8) times greater probability of increased insulin levels and a 3.0 (1.5-5.9) times greater probability for altered HOMA-IR values in the obese group compared with the overweight group (Table 3).

The simple linear correlation coefficients between the clinical, laboratory and adipokine parameters are presented in Table 4. All the parameters were significantly correlated with leptin, whereas adiponectin only exhibited a significant negative correlation with SBP. Based on these significant correlations, a multiple linear regression model was applied and adjusted for age, sex, pubertal staging, BMI, z-BMI, WC, BFP, SBP, DBP, HDL-C, TG, glucose, insulin and HOMA-IR value, considering leptin as the dependent variable. In this model, sex (*p*<0.0001), WC (*p*=0.004), BFP (*p*=0.009) and pubertal stage (*p*=0.04) explained 63% of the changes in the serum leptin levels (r^2^=0.63; leptin = -6711 + 8.39 sex +0.38 WC + 0.27 BFP – 3.92 pubertal stage). The same multiple linear regression analysis was performed with SBP as the predictor and adiponectin as the dependent variable; however, SBP exhibited no statistical significance.

In the analysis of the clinical and laboratory data, the occurrence rate of metabolic syndrome, as defined according to the adopted criteria, was 13.6% (22/161), among which there was one overweight and 21 obese individuals and 10 children under 10 years of age. Of note, children with and without metabolic syndrome exhibited similarities regarding sex, age and pubertal stage. Furthermore, all parameters in the clinical and metabolic profiles of the individuals with metabolic syndrome were significantly increased, except for LDL-C and glucose, which exhibited no statistically significant difference (Table 5).

The analysis according to the number of criteria used to characterize metabolic syndrome revealed that 32.3% (52/161) of the participants presented with none of the criteria, whereas 30.4% (49/161) presented with one criterion, 23.6% (38/161) presented with two, 11.2% (18/161) presented with three, and 2.5% (4/161) presented with four of the criteria for metabolic syndrome.

## DISCUSSION

In view of the growing body of studies on the risk factors for CVD and type 2 DM, the failure to identify and act upon these risk factors in childhood and adolescence entails the sustainment of the above pathologies into adulthood, thus increasing the probability of adverse outcomes.

In the present study, we found a high frequency (65.8%) of excess body weight reported by parents, and studies indicate that the presence of obesity in parents seems to be an important risk factor for the development of early obesity in childhood, resulting from the sum of genetic influences and the influence of the parent’s lifestyle on their children 23. In addition to obesity, a family history of DM, hypertension, dyslipidemia and early atherosclerotic disease are risk factors for CVD and DM in offspring 1,23.

Considering that the probability of permanent obesity in adulthood is 20% to 50% in cases when obesity occurs before sexual maturity and 50% to 70% in cases in which obesity occurs after the onset of puberty, the nutritional assessment of obese children and adolescents should clearly take into account chronological age and degree of sexual maturity 6. We found obesity in underage children and adolescents in equal proportions with regard to sexual maturity (49% of the pre-pubertal and 51% of the pubertal individuals). Notably, these are alarming data, and early intervention is required to avoid the comorbidities associated with obesity.

In the analysis of the studied population, we found a high frequency of increased WC (45.4%), which was more predominant in the obese group; this group also exhibited a higher BFP (total adiposity). These findings corroborate other Brazilian studies showing that increased BMI and body adiposity, particularly central adiposity, potentiate the emergence of metabolic risk factors 6,9,24. In a study of obese adolescents in São Paulo state, Brazil, Masquio et al. 10 found that WC is positively correlated with the number of metabolic syndrome parameters, inflammatory biomarkers and visceral fat and is a simple and practical method to assess visceral adiposity. Furthermore, data from the Bogalusa Heart study 25 show a high cardiometabolic risk for children with abdominal obesity and excess or normal body weight compared to children with excess body weight devoid of excess abdominal fat. In the present study, the obese group had a nine-fold greater probability of exhibiting an increased WC compared with the overweight group, thus rendering WC an important practical clinical measure to estimate cardiometabolic risk.

In a study of Brazilian adolescents ranging in age from 10 to 16 years, Moser et al. 26 have shown that triceps skin-fold thickness and BMI represent anthropometric indicators independently associated with blood pressure above P90, irrespective of abdominal adiposity and sexual maturity. Furthermore, a high BMI increased the risk of high blood pressure nearly three-fold among students with excess body weight compared with eutrophic controls. Morrison et al. 5 have shown that maintaining a normal BMI from childhood up to adulthood is a significant negative risk factor for CVD in adulthood and also that high blood pressure throughout childhood up to adulthood increases the probability of exhibiting type 2 DM. In the present study, two groups with altered BMI values were compared, and the mean blood pressure did not differ between the groups. However, the obese group exhibited a 4 times greater probability of increased blood pressure than the overweight group. In addition, we found a positive correlation between BMI, WC and blood pressure, thus reinforcing the classification of BMI and WC as anthropometric markers of cardiovascular risk.

Studies indicate that the process of atherosclerosis begins in childhood and is related to obesity and cardiovascular risk factors, such as hypertension, dyslipidemias and changes in glucose metabolism 10,11,27. In adolescents, carotid intima-media thickness, which is considered a subclinical marker of atherosclerosis, has been found to be positively correlated with parameters of metabolic syndrome, including LDL-C, low HDL-C, TG, subcutaneous abdominal fat volume, visceral fat volume and blood pressure 27,28. In the present study, changes in lipid metabolism were highly relevant, and the obese group exhibited higher means of total cholesterol, LDL-C, TG and TG/HDL-C compared with the overweight group. In the Princeton Lipid Research Clinics (LRC) follow-up study, Morrison et al. 5 showed that individuals with high TG levels from childhood up to adulthood exhibited an increase in cardiovascular events during adulthood. This result reveals the importance of early diagnosis and treatment of metabolic changes. In support of this approach, other follow-up studies have shown that overweight and obese children who became non-obese adults normalized the risks of dyslipidemia, hypertension and subclinical atherosclerosis in adulthood, thus behaving like individuals who had never been obese 29.

An increase in adipose tissue during childhood is likely an initial event in the development of alterations in glucose metabolism and represents a trigger for insulin resistance. Therefore, the search for clinical and laboratory indicators of insulin resistance in obese children is necessary 20. We found a high prevalence of acanthosis nigricans, a clinical marker of insulin resistance, and changes in glycemia, fasting insulinemia and the HOMAR-IR index values in the studied population, as previously reported in other studies of Brazilian children and adolescents 6,10,11. These changes were predominant in the obese group, except for fasting glycemia, which exhibited similar changes in both groups. The Bogalusa Heart study 30, a longitudinal study, has shown that individuals with persistently high insulin levels also exhibit higher BMI, blood pressure and levels of total cholesterol, LDL-C, TG and glucose and lower HDL-C levels compared with individuals with lower insulin levels. Furthermore, persistently high insulin levels over time were associated with a higher probability of developing hypertension, dyslipidemia and obesity in young adults. In a cohort study of white and black girls who had a diagnosis of metabolic syndrome and insulin levels within the upper quartile at 10 years of age, Morrisson et al. 31 found a high percentage of evolution into altered fasting glucose and type 2 DM 14 years later. This finding is in agreement with another study on childhood cardiometabolic risk factors and evolution into type 2 DM after 25 to 30 years 32. A significant positive association has also been found between BMI and insulin, HOMA-IR values and BMI, HOMA-IR values and WC as well as HOMA-IR values and TG among children and adolescents 6,33, thus further corroborating the results of the present study, which show a three-fold greater probability of obesity associated with increased insulin levels and HOMA-IR values.

Adipokines secreted by visceral adipose tissue are associated with components of metabolic syndrome 4,7,10. Studies on obese children, adolescents and adults have evidenced a direct correlation between leptin and adiposity markers (BMI, WC and BFP) and with glucose, insulin and HOMA-IR values 8,9,34. Additionally, studies on adiponectin have shown an inverse correlation with BMI, WC, insulin, HOMA-IR and TG values 8,35. In the present study, we found a positive correlation between leptin and adiposity markers, blood pressure, insulin and HOMA-IR values. Adiponectin was not significantly correlated with adiposity markers; however, several samples were below the detection limits of the method, and we suggest the presence of hypoadiponectinemia in these patients, which has already been documented in the literature 8,9,35. Studies have also shown that adiponectin levels decrease with age and are higher in girls 8,9. In the present work, we suggest that there was no difference between the groups, given that the overweight children were older but were predominantly female.

An increased prevalence of metabolic syndrome has accompanied the global epidemic of obesity. This increase has also occurred in the pediatric age range, and as previously observed, components of metabolic syndrome are already present at early ages. The definition of the criteria for metabolic syndrome in children and adolescents is still prone to limitations and difficulties with respect to the uniformity of the criteria used for diagnosis. However, adaptations have been used in the literature 4,6,20. In the present casuistic, we found a 13.6% occurrence rate of metabolic syndrome, and even though the criteria of the IDF are not applicable to children below 10 years of age, we found a significant number of children who met these adapted criteria. Thus, regardless of the definition of metabolic syndrome, the high percentage of individuals with at least one criterion indicates the need for early intervention regarding the detected risk factors.

The risk factors identified among obese children and adolescents tend to persist throughout adulthood if not treated, thus contributing to an ever earlier establishment of CVD. Therefore, the high percentage of clinical and metabolic changes of cardiovascular risk found in the present study on overweight and obese children and adolescents indicates the need to develop local public health policies and to establish measures involving changes in the habits and lifestyle of this population.

## AUTHOR CONTRIBUTIONS

Palhares HM developed this research and wrote the manuscript. Silva AP, Resende DC and Palhares HM performed nutritional evaluation of children and adolescents studied. Pereira GA e Palhares HM performed the statistical analysis and configured the tables. Rodrigues-Júnior V guided the assays of this research. Borges MF planned and guided this research.

## ACKNOWLEDGMENTS

We thank Prof. Dr. Vinicius Nahime de Brito of the Hormone Laboratory and Molecular Genetics (LIM 42) of the Hospital and the Faculty of Medicine of the University of São Paulo-SP for determining the leptin levels.

## Figures and Tables

**Table 1:** Clinical and laboratory characterization of overweight and obese children and adolescents recruited from the public schools of the Municipality of Uberaba, Minas Gerais State, Brazil, from February 2013 to July 2014.

Variables	Group		*p*-value
	Overweight (n=65)	Obese (n=96)	
Age (years) #	12.6±3.4	10.9±2.5	0.0002
Sex (M/F) ϕ	19/46	39/57	0.13
Pubertal stage (pre-pubertal/pubertal) ϕ	17/48	47/49	0.003
BMI (kg/m^2^) #	23.0±3.1	27.1±3.7	<0.0001
BMI z-score ¤	1.6 (1.0-2.0)	2.57 (2.0-4.9)	<0.0001
Waist circumference (cm) #	73.4±8.4	84.0±10.7	<0.0001
Body fat percentage #	34.5±9.0	43.1±8.4	<0.0001
Systolic blood pressure (mmHg) #	106.4±9.7	108.7±10.0	0.15
Diastolic blood pressure (mmHg) #	68.6±7.6	70.3±7.9	0.19
Total cholesterol (mg/dL) #	159.0±33.5	169.9±32.5	0.04
HDL-cholesterol (mg/dL) #	46.7±10.8	44.6±11.2	0.23
LDL-cholesterol (mg/dL) #	94.6±29.6	105.5±30.3	0.03
Triglycerides (mg/dL) ¤	81.0 (38.0-196.0)	91.0 (31.0-445.0)	0.04
TG/HDL¤	1.76 (0.6-5.8)	2.1 (0.6-10.3)	0.04
Glucose (mg/dL) #	85.4±12.1	87.6±11.5	0.230
Insulin (µIU/mL) ¤	11.6 (3.5-32.8)	14.5 (1.4-117.2)	<0.0001
HOMA-IR ¤	2.3 (0.7-7.1)	3.2 (0.3-28.7)	<0.0001
Leptin (ng/mL) #	15.2±9.4	24.1±11.4	<0.0001
Adiponectin (ng/mL) ¤	6.5 (0.0-96.0)	6.5 (0.0-43.2)	0.8

Source: the author

# *t*-test, values are expressed as the means ± standard deviation;

ϕ Chi-squared test;

¤ Mann-Whitney test, values are expressed as medians (Vmin-Vmax).

**Table 2:** Percentage of clinical changes in overweight and obese children and adolescents recruited from the public schools of the Municipality of Uberaba, Minas Gerais State, Brazil, from February 2013 to July 2014.

Changes	Group (%)			*p*-value	OR (95% CI)
	General (n=161)	Overweight (n=65)	Obese (n=96)		
Waist circumference §	45.3	17.0	64.6	<0.0001	9.0 (4.1-19.4)
Systolic blood pressure §	8.1	3.1	11.4	0.05	4.1 (0.9-19.0)
Diastolic blood pressure §	9.3	6.1	11.4	0.25	1.9 (0.6-6.5)
Acanthosis nigricans †	51.6	32.3	64.6	<0.0001	3.8 (2.0-7.4)
Cervical acanthosis nigricans †	44.1	18.5	61.5	<0.0001	7.0 (3.3-14.9)
Axillary acanthosis nigricans †	36.6	21.5	46.9	0.001	3.2 (1.6-6.6)

Source: the author

§ Increased;

† Present;

OR: odds ratio; 95% CI: 95% confidence interval.

**Table 3:** Percentage of laboratory changes in overweight and obese children and adolescents recruited from the public schools of the Municipality of Uberaba, Minas Gerais State, Brazil, from February 2013 to July 2014.

Changes	Group (%)			*p*-value	OR (95% CI)
	General (n=161)	Overweight (n=65)	Obese (n=96)		
Total cholesterol §	40.4	32.3	45.8	0.08	1.8 (0.9-3.4)
HDL-cholesterol ϕ	47.2	39.7	52.1	0.12	1.6 (0.9-3.1)
LDL-cholesterol §	16.5	12.7	18.9	0.29	1.6 (0.6-3.9)
Triglycerides §	23.6	15.4	29.2	0.04	2.3 (1.0-5.0)
Glucose §	10.0	9.4	10.4	0.82	1.1 (0.4-3.3)
Insulin §	36.9	23.1	46.3	0.002	2.9 (1.4-5.8)
HOMA-IR §	44.3	28.6	54.7	0.001	3.0 (1.5-5.9)

Source: the author

§ Increased;

ϕ Reduced;

OR: odds ratio; 95% CI: 95% confidence interval.

**Table 4:** Simple linear correlation (*r*) between clinical, laboratory and leptin parameters in overweight and obese children and adolescents recruited from the public schools of the Municipality of Uberaba, Minas Gerais State, Brazil, from February 2013 to July 2014.

Variable	Variable					
	BMI		WC		Leptin	
	*r*	*p*	*r*	*p*	*r*	*p*
BMI #			0.89	0.001	0.69	0.001
BMI z-score ¤	0.64	0.001	0.54	0.0001	0.47	0.0001
Waist circumference #	0.89	0.001			0.68	0.0001
Body fat percentage #	0.70	0.001	0.71	0.001	0.60	0.0001
Systolic blood pressure #	0.53	<0.0001	0.53	<0.0001	0.36	<0.0001
Diastolic blood pressure #	0.43	0.0001	0.43	0.001	0.29	0.0001
Total cholesterol #	0.09	NS	0.16	0.04	0.09	NS
HDL-cholesterol #	-0.20	0.01	-0.20	0.006	-0.10	NS
LDL-cholesterol #	0.13	NS	0.18	0.03	0.08	NS
Triglycerides ¤	0.20	0.01	0.23	0.003	0.20	0.014
TG/HDL-C ¤	0.24	0.003	0.28	0.0003	0.21	0.009
Glucose #	0.12	NS	0.14	NS	0.13	NS
Insulin ¤	0.52	<0.0001	0.50	<0.0001	0.39	<0.0001
HOMA-IR ¤	0.54	<0.0001	0.52	<0.0001	0.40	0.0001
Leptin #	0.69	0.001	0.68	0.0001		

Source: the author

# Pearsońs linear correlation coefficient

¤ Spearmańs linear correlation coefficient

NS: non-significant

**Table 5:** Clinical and laboratory characterization of individuals with and without metabolic syndrome.

Variable	METABOLIC SYNDROME		*p*-value
	NO (n=139)	YES (n=22)	
Age (years) #	11.4±3.1	11.3±2.8	0.70
Sex (M/F) ϕ	50/89	8/14	0.97
Pubertal stage(pre-pubertal/pubertal) ϕ	53/86	11/11	0.29
BMI (kg/m^2^) #	24.9±3.9	29.1±2.9	<0.0001
BMI z-score #	2.1±0.7	3.1±0.8	<0.0001
Waist circumference (cm) #	78.0±10.6	89.9±8.8	<0.0001
Body fat percentage #	37.9±8.6	50.9±8.0	<0.0001
Systolic blood pressure (mmHg) #	106.6±9.2	115.3±11.5	0.0001
Diastolic blood pressure (mmHg) #	68.4±7.2	77.6±6.9	<0.0001
Total cholesterol (mg/dL) #	163.4±32.9	179.0±33.2	0.04
HDL-cholesterol (mg/dL) #	46.9±10.6	36.6±9.7	<0.0001
LDL-cholesterol (mg/dL) #	99.4±30.3	112.1±29.3	0.07
Triglycerides (mg/dL) #	92.7±48.4	151.6±53.5	<0.0001
TG/HDL ¤	1.7 (0.6-10.4)	3.9 (2.0-9.9)	<0.0001
Glucose (mg/dL) #	86.2±11.7	90.1±11.7	0.15
Insulin (µIU/mL) ¤	12.4 (2.9-42.9)	20.6 (1.4-117.2)	0.0002
HOMA-IR ¤	2.5 (0.5-11.6)	4.3 (0.3-28.7)	0.0002
Leptin (ng/mL) #	19.3±11.1	28.7±10.8	0.0003
Adiponectin (ng/mL) #	10.7±15.1	10.1±12.1	0.86

Source: the author

# *t*-test, values are expressed as the means ± standard deviation;

ϕ Chi-squared test;

¤ Mann-Whitney test, values are expressed as medians (Vmin-Vmax).
